# DYRK1A (Dual-Specificity Tyrosine-Phosphorylated and -Regulated Kinase 1A): A Gene with Dosage Effect During Development and Neurogenesis

**DOI:** 10.1100/tsw.2006.319

**Published:** 2006-06-17

**Authors:** M. Dierssen, M. Martínez de Lagrán

**Affiliations:** Genes and Disease Program, Genomic Regulation Center, Barcelona Biomedical Research Park, 08003 Barcelona, Spain

**Keywords:** minibrain, DYRK1A, Down syndrome, monosomy 21, neurogenesis, synaptogenesis, neurodevelopment

## Abstract

DYRKs (dual-specificity tyrosine-regulated kinases) are an emerging family of evolutionarily conserved dual-specificity kinases that play key roles in cell proliferation, survival, and development. The research in the last years suggests a relevant conserved function during neuronal development, related to proliferation and/or differentiation for DYRK1A. It is expressed in neural progenitor cells and has been proposed to participate in the signaling mechanisms that regulate dendrite differentiation. In *Drosophila*, disruption of the homolog *minibrain* gene results in flies with reduced neuroblast proliferation, decreased numbers of central brain neurons, and learning/memory deficits. Knockout DYRK1A mice are embryonic lethal, and heterozygotes show decreased viability and region-specific reductions in brain size. In humans, DYRK1A has been proposed to be involved in the neurodevelopmental alterations associated with Down syndrome. The large number of protein interaction and putative substrates described for DYRK1A suggest multiple pathways and functions to be involved in its developmental function. This review focuses on the functional role that DYRK1A plays in brain development.

## INTRODUCTION

DYRK1A is a member of the DYRK (dual-specificity tyrosine[Y]-regulated kinase) family. DYRKs constitute an evolutionarily conserved family of proline- or arginine-directed protein kinases belonging to the CMGC family of cyclin-dependent kinases (CDKs), mitogen-activated protein kinases (MAPKs), GSK, and CDK-like kinases (CLKs)[1,2,3]. The DYRK family has multiple members that can be predominantly nuclear (DYRK1A and DYRK1B) or cytoplasmic (DYRK2–4 and homeodomain interacting protein kinase 3 [HIPK3]/DYRK6)[1]. RT-PCR and western blotting suggested that DYRK1A

and DYRK2 were major representatives of nuclear and cytoplasmic DYRKs, respectively, in Jurkat T cells[4].

DYRKs are evolutionarily conserved and play key roles in cell proliferation, survival, and development[5]. As its name implies, the DYRK1A enzyme has dual substrate specificities; autophosphorylation for self-activation takes place on the tyrosine-321 residue in the active loop of the catalytic domain[3,6], and target protein phosphorylation occurs on serine/threonine residues. Dual-specificity protein kinases play pivotal regulatory roles in the signaling of cell proliferation and development. One well-studied example is mitogen-activated protein (MAP) kinase kinases (or MEKs) in which downstream targets of their S/T and Y phosphorylation (e.g., ERKs JNK, P38) mediate cell growth, survival, and/or differentiation. By comparison, DYRK kinases are a recently discovered subfamily of MEK- and MAPK-related kinases with unique N- and C- terminal domains that may regulate specific pathway (and partner) interactions. In fact, the ability of DYRK1A, SNF1LK, RIP4, and DSCR3p to interact and act *in vivo* as a mitogen-activated kinase cascade activating the 7MSR effector pathways has been described[7]. DYRK1A has been reported to phosphorylate or interact with several proteins, including STAT3, FHKR, Gli-1, eIF2Be, Tau, dynamin, glycogen synthase, 14-3-3, CREB, cyclin L2, Arip4, Hip-1, PAHX-AP1, and SF3b1. Its diverse array of interactions suggests that DYRK1A participates in multiple biological pathways[8,9]. Besides, its expression pattern suggests that it may play differential functions during development and in the adult. DYRK1A mRNA expression has been detected in the adult and at different development stages by northern blot and *in situ* hybridization, in both human and murine tissues[10,11,12,13,14,15]. These studies have shown a generalized expression of DYRK1A transcripts in all tissues considered including the brain at different developmental stages. In the adult brain, DYRK1A distribution shows specific expression in selected areas of the brain[16,17].

However, possibly the most relevant *in vivo* function of DYRK1A is related to neurodevelopment, being crucial for cell proliferation and differentiation in specific brain areas, as will be reviewed below.

## DYRK1A HOMOLOGS IN INVERTEBRATES HAVE A ROLE IN NEUROGENESIS

The role of DYRK1A in neurodevelopment was first deduced from the function of its *Drosophila* orthologue, the minibrain (mnb) gene for which four alleles have been described. Protein extracts of embryos and pupae contain consistently more mnb protein than those of adults[18]. In late embryos, mnb mRNA is expressed in the ventral cord and in the brain, but not in the peripheral nervous system and anti-mnb antibodies stain most prominently the mushroom body neuropil and the optic lobes. Thus, mnb appears to be expressed prominently in the larval tissue where neuronal progeny are generated during postembryonic development. Strikingly, in the adult, the level of protein is low in optic lobes and central brain hemispheres, but relatively high in retinal pigment cells and in the alpha, beta, and gamma lobes and peduncle of the mushroom bodies[18], suggesting a different functional role during development and in the adult brain.

*Drosophila* mnb is required for normal postembryonic neurogenesis. Flies that carry mutations in the mnb gene express at 30–60% mnb protein level compared to that of wild types, and display a specific and marked size reduction in specific brain areas as well as behavioral deficiencies[18]. The external appearance of mutant flies, including body and sensory organs, is nearly indistinguishable from wild type. The brains of adult mutant flies are reduced in size, but show no gross alterations in neuronal architecture. The central brain hemispheres are reduced mainly in their ventral to dorsal and anterior and posterior extensions. Axon bundles that project from the lobula complex to the lateral protocerebrum (optic stalk) are visibly thinner in the mutants, and the number of anterior optic tract fibers and of cervical connective fibers is reduced. The proposed mechanism underlying this size reduction might be related to the abnormal spacing of neuroblasts in the outer proliferation center of larval brain caused by the mutation, with the implication that mnb fly neuroblasts produce less neuronal progeny than do wild type. Furthermore, this insufficient number of distinct neurons in mnb brains might be responsible for the specific abnormalities in visual and olfactory behavior, in the absence of eye and antennal morphology abnormalities[18]. Moreover, freely walking mutant flies show reduced locomotor activity and odor discrimination is poor[18].

DYRK kinases have been identified in all eukaryotic organisms examined to date. In *Saccharomyces cerevisiae*, Yak1p is the protein kinase with the highest sequence similarity with mnb and DYRK1. Yak1p is a dual-specificity protein kinase that autophosphorylates on Tyr-530 and phosphorylates exogenous substrates on Ser/Thr residues[19]. It functions as a negative regulator of the cell cycle in *S. cerevisiae*, acting downstream of the RAS/protein kinase A[20]. YakA appears to regulate the transition from growth to development in *Dictyostelium*, causing cell-cycle arrest in nutrient-rich medium and promoting developmental events such as the expression of genes required for cyclic AMP signaling[21].

In the genome sequence of *Caenorhabditis elegans*, three DYRK/mnb-like genes, termed mbk-1, mbk-2, and hpk-1, were identified. These genes are widely expressed and localize to distinct subcellular compartments. Deletion of mbk-1, the gene most closely related to DYRK1A, causes no obvious defects, while additional copies of the mbk-1 gene produce behavioral defects in chemotaxis correlated with mbk-1 gene dosage[22]. Conversely, mbk-2 is essential for viability[22] and has a role in asymmetric cell division in *C. elegans*. In the newly fertilized *C. elegans* zygote, cytoplasmic determinants become localized asymmetrically along the anterior-posterior axis of the embryo. The mitotic apparatus then orients so as to cleave the embryo into anterior and posterior blastomeres that differ in both size and developmental potential. In mbk-2 mutants, the initial mitotic spindle is misplaced and cytoplasmic factors, including the germline-specific protein PIE-1, are mislocalized. These findings support a role of mbk-2 in the regulation of spindle positioning during cell division and in the localization of cytoplasmic determinants[23].

## ROLE OF DYRK1A IN VERTEBRATE DEVELOPMENT

In chicken early embryos, DYRK1A is expressed before the onset of neurogenesis in the three locations where neuronal precursors are originated: neuroepithelia of the neural tube, neural crest, and cranial placodes. It has been proposed that DYRK1A defines a transition step between proliferating and neurogenic divisions of neuroepithelial progenitor cells[12]. DYRK1A is transiently expressed during a single cell cycle of these progenitor cells preceding and overlapping with the expression of Tis21, an antiproliferative gene expressed in the onset of neurogenic divisions. A second wave of DYRK1A expression takes place in the brain of intermediate and late embryos restricted to certain populations of neurons, but not astroglial or oligodendroglial cells. Interestingly, DYRK1A expression takes place at the time of dendritic tree differentiation and is initiated by a transient translocation from the cytoplasm to the nucleus. Afterwards, DYRK1A protein is transported to the growing dendritic tree, where it colocalizes with dynamin 1[24], a GTPase putative substrate of DYRK1A that plays a fundamental role in synaptic vesicle recycling, clathrin-mediated endocytosis, intracellular membrane trafficking[25], and neurite outgrowth[26,27]. Moreover, in different model systems, it has been proposed that DYRK1A might also have a role in shaping the interaction of the spine membrane with the actin cytoskeleton. Yang and co-workers[28] showed that overexpression of a kinase-deficient form of DYRK1A attenuates the neurite outgrowth induced by a neurogenic factor in immortalized hippocampal cells. Finally, during the neuronal differentiation of embryonic hippocampal neuroprogenitor cells, DYRK1 selectively binds to and phosphorylates huntingtin-interacting protein 1 (Hip-1), a proapoptotic mediator. The DYRK1-mediated phosphorylation of Hip-1, in response to bFGF, results in the blockade of Hip-1–mediated neuronal cell death as well as the enhancement of neurite outgrowth[29]. These data suggest that DYRK1 has a dual function in regulating neuronal differentiation and proliferation.

### The Functional Roles of Mammalian DYRK1A

The mammalian DYRK1A are highly conserved, and the predicted human and murine DYRK1A proteins both contain nuclear targeting signal sequences, a protein kinase domain, a putative leucine zipper motif, and a highly conserved, 13-consecutive-histidine repeat[13,14]. It has been proposed that DYRK1A participates in mammalian brain development due to the fact that it is highly expressed in brain during development[15,17]. *In situ* hybridization of DYRK1A to mouse embryos (13, 15, and 17 days postcoitus) indicates a specific spatial and temporal pattern of expression, with the most abundant signal localized in brain gray matter, spinal cord, and retina[14]. The expression of DYRK1A protein gradually decreases to low levels at adult stage, being expressed in selected regions of the cerebrum, cerebellum, and brain stem in the adult rat central nervous system[15]. Mouse DYRK1A is expressed in brain areas controlling motor function, such as the granular layer of the cerebellar cortex, deep cerebellar nuclei, motor nuclei of the medulla and pons, the gray matter of the spinal cord, and subcortical areas[17] (Fig. 1).

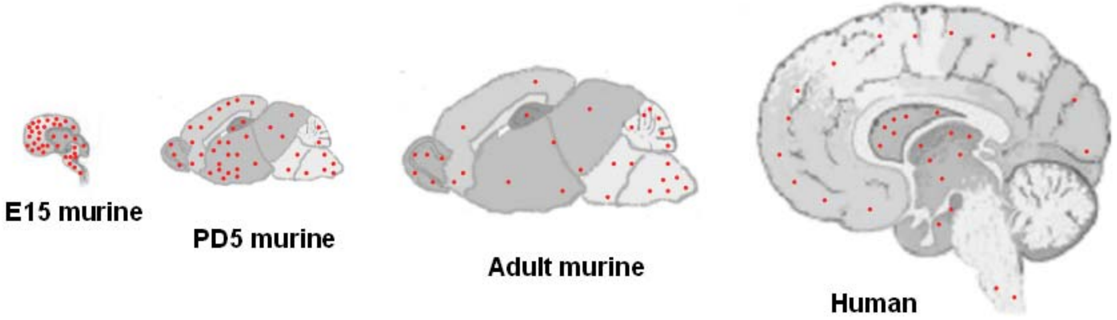

**FIGURE 1.** Spatial and temporal distribution of DYRK1A during life span. DYRK1A is abundant in early developmental stages in neurons and has an expression pattern that appears in two waves in vertebrate embryos. The first wave is a transient expression in neural progenitor cells of early embryos[12]. The second wave occurs during late embryonic development. High levels of expression are still detected in early postnatal life that decrease until adulthood. In the adult, DYRK1A immunostaining is restricted to specific brain areas. It is strong in the olfactory bulb, the cerebellar cortex and functionally related structures, the spinal cord, and most of the motor nuclei of the midbrain and brain stem. These data agree with a possible implication of this kinase in the physiology of olfaction and motor functions.

Subcellular expression studies in primary cultures of the cerebellum have shown the presence of DYRK1A in the nucleus and cytoplasm of neurons and astrocytes, and in both the nuclear and cytoplasm-enriched fractions in whole adult brain homogenates. Studies in postmortem tissues of the developing and adult human brain also showed DYRK1A expression in both cell nucleus and cytoplasm of neurons[30]. The subcellular localization of the protein seems to be of crucial importance to some of its functions. During embryonic development, DYRK1A expression in vertebrates takes place in two waves, the first occurring transiently in neural progenitor cells during the transition from proliferating to neurogenic divisions, and the second in mid- and late embryos at the time of dendritic tree differentiation. This second expression wave is initiated by a transient translocation from the cytoplasm to the nucleus, suggesting its involvement in the signaling mechanisms that trigger the final steps of neuronal differentiation. Afterwards, DYRK1A protein is transported to the growing dendritic tree.

Moreover, an increased DYRK1A accumulation presented in corpora amylacea in the memory and motor system subdivisions in aged humans indicates that Mnb/DYRK1A could be colocalized with neuron degeneration[30]. This assumption is further supported by the fact that the expression of DYRK1A in the nucleus is augmented in particular disease-dependent, sensitive neurons[31]. Whether DYRK1A translocation from the cytoplasm to the nucleus in disease is associated with some positive effect on dendrites, as suggested during development[12], is not known.

## *In Vivo* Evidence for Dosage Effects of Mammalian DYRK1A

### *Effects of DYRK1A Deletion*

To date there is only one DYRK1A null mice created by gene targeting[32], with no conditional models available. Homozygous null DYRK1A mutant embryos (DYRK1A-/-) present delayed general growth with an overall reduction in organ growth, including the developing brain where neurogenesis appears to be decreased. Moreover, these DYRK1A-/- mice die during midgestation. Mice heterozygous for the mutation (DYRK1A+/- mice) show decreased neonatal viability and reduced body size from birth to adulthood. Neurobehavioral analysis revealed preweaning developmental delay of heterozygous mice and specific motor[33] and learning[32] deficits in adults. Brains of heterozygous mice are decreased in size in a region-specific manner, although the cytoarchitecture and neuronal components in most areas are not altered, as was also described for *Drosophila* mutants. Cell counts showed increased neuronal densities in some brain regions and decreases in the number of neurons in the superior colliculus, which exhibited a significant size reduction. The reduction in brain size and behavioral defects observed provide evidence about the nonredundant vital role of DYRK1A and suggest a conserved mode of action that determines normal growth and brain size in both mice and flies[32].

More specifically, alterations in the phenotype of neocortical pyramidal cells are observed in the DYRK1A+/- mouse[34]. Basal dendritic arbors of layer III pyramidal cells of DYRK1A+/- mice are smaller, less branched, and less spinous than those in their control littermates. It remains to be determined whether pyramidal cells in juvenile DYRK1A+/- mice do not achieve the structural complexity of those in wild type mice or whether they undergo more intense dendrite retraction during maturation. DYRK1A might be implicated in both processes since it has been shown that overexpression of a kinase-deficient DYRK1A impedes neurite outgrowth[28] and that a putative substrate of DYRK1A, dynamin 1, is also implicated in neurite outgrowth[26], as describe above. Since pyramidal cells comprise more than 70% of the neurons in the cortex, it is reasonable to think that DYRK1A is involved in the determination of complexity of circuits in the mature cerebral cortex[35].

### *In Vivo Overexpression of DYRK1A*

Different research groups during the last years have developed mice that overexpress DYRK1A. These previous studies seem to confirm that normal amounts of the DYRK1A gene are necessary for normal brain development in the fetus, so that either reduction or excess dosage of the gene interferes with normal brain development. In early experiments, Smith et al.[36], using Down syndrome as a model for complex trait analysis, sought to identify loci from 21q22.2 which, when present in an extra dose, could contribute to learning abnormalities. Their strategy was the creation of panels of YAC transgenic mice propagating targeted megabase regions of the genome. Such a panel of mice was called an "*in vivo* library" and was used to identify candidate genes, based on functional screens of members of the library. Smith and colleagues generated low-copy number transgenic mice, containing 4 different YACs that together cover approximately 2 Mb of contiguous DNA from 21q22.2.

Independent mouse lines derived from each of these YAC transgenes were subjected to a series of behavioral and learning assays. One of these lines, a 570-kb YAC, showed distinct learning deficits compared with the other 21q22 YAC transgenics and nontransgenic control animals. The interval responsible for these defects was narrowed to a 180-kb critical region as a consequence of YAC fragmentation (152F7 line), a fragment that in addition to genes also located on the other YACs, bears the DYRK1A gene. The phenotype of the 152F7 line was recently further analyzed[37], showing learning impairment and hyperactivity during development, together with increased brain weight and neuronal size. At a biochemical level, DYRK1A overexpression was associated with an increase in phosphorylation of the transcription factor FKHR and with high levels of cyclin B1, suggesting a correlation *in vivo* between DYRK1A overexpression and cell cycle protein alteration. In addition, altered phosphorylation of transcription factors of CREB family was observed, supporting a role of DYRK1A overexpression in the neuronal abnormalities seen in Down syndrome and suggesting that this pathology is linked to altered levels of proteins involved in the regulation of cell cycle.

Some years after the initial generation of YAC transgenics overexpressing DYRK1A, and based on the hypothesis that the overexpression of a single gene may have per se an impact on development and brain function, Altafaj et al.[38] generated transgenic mice overexpressing the full-length cDNA of DYRK1A. Transgenic DYRK1A mice exhibited delayed craniocaudal maturation with functional consequences in neuromotor development and general psychomotor delay. DYRK1A mice also showed altered motor skill acquisition and hyperactivity, which was maintained to adulthood[38,39]. In different learning paradigms, DYRK1A mice showed a significant impairment in spatial learning and cognitive flexibility, indicative of hippocampal and prefrontal cortex dysfunction. Regarding activity levels, TgDYRK1A mice present with a mild hyperactive phenotype that is only observed in novelty-related situations, but not in basal conditions[38], whereas YAC transgenic mice containing human DYRK1A (TgYAC157F, see above) show normal levels of activity or even slight hypoactivity[36]. These results, together with the expression of DYRK1A in different areas of the brain related to motor function[17], suggest a relationship between DYRK1A expression levels and the neuromotor development and motor activity and competence in the adult. In line with this, mice hemizygous for the targeted mutation of DYRK1A allele (DYRK1A+/- mice)[32] exhibit a significant decrease in motor horizontal and vertical activity and poor motor control that could be linked by an abnormal function of specific motor systems in the brain. Moreover, the alterations in motor parameters observed in Down syndrome, also support this hypothesis.

The latest DYRK1A transgenic mice have been generated very recently by Ahn et al.[40] using a bacterial artificial chromosome (BAC) clone. The advantage of this model is that the BAC contained only the complete DYRK1A genomic DNA fragment, which included the endogenous human promoter giving rise to about a 1.5-fold overexpression of DYRK1A (mouse DYRK1A plus human DYRK1A) protein compared to nontransgenic control littermates. Also, the BAC TG mice contained only one copy of the human DYRK1A gene integrated into its genome. However, the BAC TG mice phenotype show differences with all previously generated DYRK1A overexpressing mice. Factors such as the different genetic background, the age of the mice, or the fact that a human DYRK1A was contained in the BAC have been claimed by the authors to account for the differences observed. For example, BAC TG present much more severe learning and memory deficiencies in a Morris water maze test than all previously available models. However, the measures taken in the analysis of the learning pattern (cumulative distance to the platform, instead of escape distance or latencies) are not easily comparable with the previously obtained results, and thus it is difficult to ensure that the impairment is more severe or not than that observed in the previous DYRK1A transgenic models. In fact, electrophysiological analyses in BAC TG mice revealed increases in long-term potentiation (LTP) somewhat difficult to conceal with the severe deficits in learning, although this result was explained by the decreased long-term depression (LTD), giving rise to an altered bidirectional synaptic plasticity[41] that could be responsible for the learning defects observed.

## SUBSTRATES AND INTERACTORS FOR DYRK1A

DYRK1A has been reported to phosphorylate a myriad of substrates both located in the nuclear and cytoplasmic compartments, thus suggesting that the subcellular localization of the protein may determine its functional role in the cell (Table 1). However, only a reduced number has been proven *in vivo*. In this regard, it should be born in mind that the interactions of proteins and, therefore, their physiological relevance depend on their coexpression in time and place. To this end, the information available is often limited and inconsistent in the tissues, cell types, or developmental ages that are examined.

**TABLE 1**
**Nuclear and Cytoplasmic Interactors and Substrates of DYRK1A, Subcellular Localization, and Physiological Relevance**

| SUBSTRATE | SUBCELLULAR LOCALIZATION | FUNCTION | REFERENCE |
|---|---|---|---|
| 14 -3 -3 | nuclear/ cytosolic | Regulation of scaffolding, protection of proteolysis, dephosphorylation, and sequestration of target protein | Kim et al. (2004) |
| Androgen receptor-interacting protein 4 (Arip4) | nuclear | Regulation of steroid hormone-induced transcription | Sitz et al. (2004) |
| cAMP responsive element binding protein (CREB) | nuclear | Neuronal development and differentiation<br>Synaptic plasticity<br>Learning and memory | Yang et al. (2001) |
| Cyclin L2 | nuclear | Transcriptional regulation involved in pre-mRNA splicing | De Graaf et al. (2004) |
| Dynamin | cytosolic | Endocytic synaptic vesicle recycling | Chen-Hwang et al. (2002)<br>Huang et al. (2004) |
| Eukaryotic initiation factor 2Bepsilon (eIF2Bε) | cytosolic | Regulation of the initiation of mRNA translation | Woods et al. (2001a) |
| Forkhead (FKHR) | nuclear | Control of gene expression by insulin and regulation of apoptosis by survival factors. | Woods et al (2001b)<br>von Groote-Bidlingmaier et al. (2003) |
| Glioma-associated oncogene (GLI1) | nuclear | Transcriptional regulation | Mao et al. (2002) |
| Glycogen synthase | cytosolic | Regulation of glucose metabolism | Skurat & Dietrich (2004) |
| Huntingtin interacting protein-1 (Hip-1) | cytosolic | Potential role in endocytosis and the maintenance of actin cytoskeleton | Kang et al. (2005) |
| Nuclear factor of activated T cells (NFAT) | nuclear/ cytosolic | Regulation of development, cell differentiation and cell adaptation | Gwack et al. (2006) |
| Phytanoyl-CoA α-hydroxylase associated protein 1 (PAHX-AP1) | cytosolic | Unknown<br>Possibly related to Refsum disease | Bescond & Rahmani (2005) |
| Ras/MAP kinase pathway | nuclear/ cytosolic | Cell growth, differentiation, and transformation | Kelly & Rahmani (2005) |
| SF3b1 | nuclear | Regulation of pre mRNA-splicing | de Graaf et al. (2006) |
| Signal transducer and activator of transcription 3 (STAT3) | nuclear | Activation of transcription | Matsuo et al. (2001) |
| tau | cytosolic | Regulation of microtubule dynamics<br>Hyperphosphorylated in Alzheimer's disease | Woods et al. (2001a) |

DYRK1A activity is dependent on the autophosphorylation of a conserved tyrosine residue in the activation loop[6,42], an essential maturation event required for full enzyme activity. However, the molecular mechanism by which this happens is unknown. Autophosphorylation of the critical activation-loop tyrosine is intramolecular and mediated by the nascent kinase passing through a transitory intermediate form. This DYRK intermediate differs in residue and substrate specificity, as well as sensitivity to small-molecule inhibitors, compared with its mature counterpart. The intermediate's characteristics are lost on completion of translation, making the critical tyrosine autophosphorylation a “one-off” inceptive event[3].

The yeast two-hybrid approach has been used to identify binding partners of DYRK1A. Using this method, the interaction of DYRK1A with 14-3-3, a protein implicated in several cell functions as the regulation of scaffolding, protection of proteolysis, dephosphorylation, and sequestration of target protein has been described[43]. In the presence of Mg-ATP, DYRK1A can inactivate glycogen synthase by phosphorylation, suggesting a possible role of this kinase in glycogen synthesis regulation[44]. The C-terminal region of DYRK1A interacts with a brain-specific protein, phytanoyl-CoA alpha-hydroxylase-associated protein 1 (PAHX-AP1, also named PHYHIP), which interacts with phytanoyl-CoA alpha-hydroxylase (PAHX, also named PHYH), a Refsum disease gene product. In PC12 cells cotransfected with both plasmids, DYRK1A intracellular localization was shown to change from the nucleus to the cytoplasm in the presence of PAHX-AP1[45], suggesting that subcellular localization of DYRK1A may define distinct cellular functions of the protein. In this regard, the protein kinase is distributed throughout the nucleoplasm, accumulating in speckle-like regions. It has been proposed[46] that the histidine-rich region represents a novel splicing speckle-targeting signal. Moreover, overexpression of DYRK1A induced DYRK1A activity-specific speckle disassembly. Thus DYRK1A protein kinase may play a role in regulating the biogenesis of the splicing speckle compartment.

Most of the DYRK1A nuclear substrates are transcription factors that generally show enhanced activity on phosphorylation[47,48,49], thus suggesting that this protein is involved in regulation of gene expression. DYRK1A modulates the activity of the c-AMP response element binding protein (CREB), which participates in signal transduction pathways involved in synaptic plasticity and neuronal differentiation[28]. Moreover, the fact that DYRK1A can phosphorylate cyclin L2 protein[50] might indicate its role in cell cycle regulation. Other known substrates of DYRK1A include the transcription factors GLI1 or FKHR[51,52]. More recently, Sitz et al.[53] have shown that DYRK1A acts synergistically with the androgen receptor interacting protein, ARIP4, to enhance transcriptional activity of glucocorticoid receptor, although in this case, the kinase activity of DYRK1A is not required. Gwack et al.[4] have shown that DYRKs are novel regulators of NFAT. DYRK1A and DYRK2 counter calcineurin-mediated dephosphorylation of NFAT1 by directly phosphorylating the conserved serine-proline repeat 3 (SP-3) motif of the NFAT regulatory domain, thus priming further phosphorylation of the SP-2 and serine-rich region 1 (SRR-1) motifs by GSK3 and CK1, respectively. Finally, DYRK1A can phosphorylate *in vitro* and *in vivo* the splicing factor SF3b1 [54], in which phosphorylation is increased during splicing catalysis[55] and in mitosis[56], suggesting that DYRK1A may be involved in the regulation of pre-mRNA-splicing.

In the cytoplasm, DYRK1A phosphorylation of dynamin 1 (see above) regulates the interaction of this protein with amphiphysin 1 and endophilin 1, thus suggesting that DYRK1A may function as a regulator controlling the assembly of endocytic apparatus since the interactions of dynamin with amphiphysin and endophilin are essential for the formation of endocytic complexes[26,57]. DYRK1A overexpression potentiates nerve growth factor (NGF)–mediated PC12 neuronal differentiation by up-regulating the Ras/MAP kinase signaling pathway independently of its kinase activity[58]. Furthermore, DYRK1A prolongs the kinetics of ERK activation by interacting with Ras, B-Raf, and MEK1 to facilitate the formation of a Ras/B-Raf/MEK1 multiprotein complex, indicating that DYRK1A may play a critical role in Ras-dependent transducing signals that are required for promoting or maintaining neuronal differentiation.

However, additional roles for DYRK1A have to be proposed, as its expression is still prominent in the adult brain. Of interest to human neurodegenerative pathology, DYRK1A phosphorylates human microtubule associate protein tau at Thr212 *in vitro*, a residue that is phosphorylated in fetal tau and hyperphosphorylated in Alzheimer’s disease and tauopathies[48], and it has been postulated that

DYRK1A might be involved in age-associated degeneration of astrocytes and neurons[30]. Phosphorylation of Thr212 primes tau for phosphorylation by GSK-3 at Ser208, a serine that is modified in Alzheimer's disease[59]. In this regard, increased DYRK1A immunoreactivity in the cytoplasm and nuclei of scattered neurons of the neocortex, entorhinal cortex, and hippocampus in Alzheimer's disease and Down syndrome with associated Alzheimer`s disease has been recently described[31]. DYRK1A is found in sarkosyl-insoluble fractions, which are enriched in phosphorylated tau in Alzheimer's disease brains, a pattern that occurs for other kinases related with tau phosphorylation *in vitro*[31], thus suggesting a possible association of DYRK1A with neurofibrillary tangle pathology.

The biological significance of these interactions remains to be established. DYRK1A has overlapping substrate/target protein specificities, with several proteins such as Sumo3, NRIP1, and the transcription factor Gabinding protein alpha subunit (GABPA)[60]. The predicted consequences of interactions of these genes are complex and diverse, and involve altered levels of post-translational modification, altered stoichiometry, competition, and cascade effects.

## CONCLUSION

The data summarized here suggest a relevant function for DYRK1A during neuronal development, related to both proliferation and differentiation processes. It is expressed in brain areas where neuronal progeny are generated and is necessary for the correct development of specific brain areas where it might be affecting cell cycle and proliferative events. This neurogenic function is dosage dependent, so that cell numbers in specific brain regions are altered both by reduced or enhanced levels of the protein. Its role in differentiation is even more important. It has been reported to influence neurite outgrowth induced by a neurogenic factor in immortalized hippocampal cells and modulates the activity of signal transduction pathways involved in synaptic plasticity and neuronal differentiation. Moreover, DYRK1A is transported through the growing dendritic tree where it colocalizes with dynamin. The relevance of DYRK1A in neurogenesis, and thus in neurodevelopmental disorders such as Down syndrome has provoked the search of molecules that could inhibit its expression and be possible therapeutic targets.

## ACKNOWLEDGMENTS

The authors want to acknowledge the Jerôme Lejeune Foundation for their continuous support to Down syndrome research.
